# Transforming Surgical Training With AI Techniques for Training, Assessment, and Evaluation: Scoping Review

**DOI:** 10.2196/58966

**Published:** 2025-11-18

**Authors:** David Escobar-Castillejos, Ari Y Barrera-Animas, Julieta Noguez, Alejandra J Magana, Bedrich Benes

**Affiliations:** 1 Facultad de Ingeniería Universidad Panamericana Ciudad de México Mexico; 2 Facultad de Ingeniería Universidad Nacional Autónoma de México Ciudad de México Mexico; 3 School of Applied and Creative Computing and School of Engineering Education Purdue University West Lafayette, IN United States; 4 Department of Computer Science Purdue University West Lafayette, IN United States

**Keywords:** artificial intelligence, technology-enhanced learning, simulation-based training, performance assessment, medical training, surgery, higher education, educational innovation

## Abstract

**Background:**

Artificial intelligence (AI) has introduced novel opportunities for assessment and evaluation in surgical training, offering potential improvements that could surpass traditional educational methods.

**Objective:**

This scoping review examines the integration of AI in surgical training, assessment, and evaluation, aiming to determine how AI technologies can enhance trainees’ learning paths and performance by incorporating data-driven insights and predictive analytics. In addition, this review examines the current state and applications of AI algorithms in this field, identifying potential areas for future research.

**Methods:**

Following the PRISMA-ScR (Preferred Reporting Items for Systematic Reviews and Meta-Analyses extension for Scoping Reviews) guidelines, the PubMed, Scopus, and Web of Science were searched for studies published between January 2020 and March 18, 2024. Eligibility criteria included English-language full-text articles that investigated the application of AI in surgical training, assessment, or evaluation; non-English texts, reviews, preprints, and studies not addressing AI in surgical education were excluded. After duplicate removal and screening, 56 studies were included in the analysis. Data were structured by categorizing studies according to surgical procedure, AI technique, and training setup. Results were synthesized narratively and summarized in frequency tables.

**Results:**

From 1400 initial records, 56 studies met the inclusion criteria. Most were journal articles (84%, 47/56), with the remainder being conference papers (16%, 9/56). AI was most frequently applied in minimally invasive surgery (27%, 15/56), neurosurgery (20%, 11/56), and laparoscopy (16%, 9/56). Common techniques included machine learning (20%, 11/56), clustering (14%, 8/56), deep learning (11%, 6/56), convolutional neural networks (11%, 6/56), and support vector machines (11%, 6/56). Training setups were dominated by simulation platforms (33%, 19/56) and box trainers (24%, 13/56), followed by surgical video analysis (16%, 9/56), and robotic systems such as the da Vinci platform (13%, 7/56). Across studies, AI-enhanced training environments provided automated skill assessment, personalized feedback, and adaptive learning trajectories, with several reporting improvements in trainees’ learning curves and technical proficiency. However, heterogeneity in study design and outcome measures limited comparability, and algorithmic transparency was often lacking.

**Conclusions:**

The application of AI in surgical training demonstrates the potential to enhance skill acquisition and support more efficient, personalized, and adaptive learning pathways. Despite encouraging findings, several limitations exist, including small sample sizes, the lack of standardized evaluation metrics, and insufficient external validation of AI models. Future studies should aim to clarify AI methodologies, improve reproducibility, and develop scalable, simulation-based solutions aligned with global education goals.

## Introduction

Scientific advances have significantly influenced the evolution of education and training in recent decades. Emerging technologies such as technology-enhanced learning and simulation-based training have played a crucial role in improving the learning experience of practitioners and have become essential in modern education systems [1].

Traditionally, surgical training has mainly focused on gaining experience through a significant number of surgeries and direct involvement, in which trainees receive less supervision from experienced surgeons as they gain competence and eventually become capable of doing surgeries independently [2]. This model embodies the “see one, do one, teach one” approach [3]. An experienced surgeon first executes a procedure, which the trainee observes. Then, under supervision, the trainee replicates the process. Finally, upon achieving competence, the trainee is expected to instruct others on how to perform it. This approach underscores the importance of direct observation, practical experience, and the ability to transmit information and expertise to future generations of medical practitioners. However, it also raises inquiries regarding the diversity of learning experiences, the consistency of the skills acquired, and the stress that it places on seasoned surgeons and trainees to quickly comprehend and transmit complex procedures involving inherent risks [4,5]. Acquiring and improving skills in the field of medicine are complex processes that last throughout a physician’s career. Since the 1990s, ongoing discussions have focused on enhancing teaching practices [6].

Researchers have developed various simulators and training platforms to address these challenges and the demands of an expanding spectrum of surgical operations [7]. These tools enable trainees to develop expertise in different surgical procedures and provide the benefit of unlimited practice opportunities, customizable difficulty levels, and cost-effective solutions that emulate the difficulties of actual surgery procedures [8,9]. Furthermore, these platforms offer a secure and interactive setting that promotes learning through experimentation, enabling risk-free practice. Nevertheless, there remains considerable potential to improve the effectiveness of these training setups [10,11].

As technological advancements continue, interest in incorporating artificial intelligence (AI) into medical training has also increased [12]. AI, with its capacity to emulate certain aspects of human cognition, has the potential to enhance educational outcomes and transform traditional methods of training and teaching [13]. It enables the creation of the next generation of autonomous systems to execute tasks usually performed by individuals, representing a substantial advancement in computer science. Furthermore, AI algorithms could assist in enhancing conceptual understanding, facilitating virtual practice, and offering analytical feedback on performance. Through the use of data-driven insights and predictive analytics, AI has the potential to revolutionize surgical training, offering customized and efficient learning pathways.

This scoping review aims to map and analyze current applications of AI in surgical training, assessment, and evaluation, identifying the most common surgical procedures, AI techniques, and training setups while highlighting gaps and opportunities for future research. The following research questions guided this study:

1. What are the specific surgical procedures where AI algorithms are most frequently applied in surgical training?
2. Which AI techniques have been used in surgical training and evaluation?
3. How are AI techniques being used to assess and improve surgical training?
4. How do AI applications in surgical training affect the learning curve of surgical residents and fellows?

The paper is organized as follows: the “Methods” section outlines the methodology used to carry out this scoping review. The “Results” section provides a comprehensive overview of the findings, shows additional findings, and identifies potential areas for opportunity. The “Discussion” section presents an outline of the research questions, shows additional findings, identifies potential areas for opportunity, acknowledges the limits of the current review, and concludes with final thoughts and directions for future research in the realm of AI in surgical education.

Although there are different definitions and approaches to what AI is, this study is particularly interested in Russell and Norvig’s [14] approach to systems that act rationally, that is, systems that act intelligently and rationally, ideally in the best possible way given the available information. AI is a disruptive technology that is reshaping education, facilitating a shift toward more efficient teaching protocols [15]. It enables machines to imitate various complex human skills, and AI-based techniques are typically employed in the following areas:

- Expert systems “emulate the behavior of a human expert within a well-defined, narrow domain of knowledge” [16].
- Intelligent tutoring systems emulate “model learners’ psychological states to provide individualized instruction. They… help learners acquire domain-specific, cognitive, and metacognitive knowledge” [17].

AI can be subdivided into machine learning (ML), which further includes deep learning (DL). ML aims to “perform intelligent predictions based on a data set” [18]. It uses statistical, data mining, and optimization methods to design models that can identify patterns and make predictions with higher precision than human experts. In this field, there are 3 fundamental ML paradigms:

- Supervised learning uses input data and their matching labeled output to train models [19]. A labeled output is data that has been assigned labels to add context; consequently, the objective of supervised learning is to learn and predict outputs for unseen data based on the initial input-output pairs.
- Unsupervised learning involves working with unlabeled data [20]. The algorithms autonomously attempt to discern patterns and relationships within the data.
- Reinforcement learning uses an autonomous entity known as an agent, which learns to make decisions by performing activities inside an environment to reach a specific objective [21]. The feedback the agent receives in the form of rewards or penalties serves as a guide as it iteratively refines its strategy to achieve optimal performance.

Finally, DL is a branch of machine learning that uses artificial neural networks to replicate the sophisticated processes of the human brain [22]. Algorithms in this category learn to identify patterns and comprehend large datasets. DL is highly efficient because it can automatically extract and learn high-level characteristics from data, reducing the need for manual feature selection. It excels at handling complex tasks such as image and audio recognition, natural language processing, image generation, and data-driven prediction.

Numerous models have been developed within AI to address challenging problems and tasks across different sectors and research fields. Each approach provides certain advantages specific to the type of data to be processed and the analytical needs (see Textbox 1).

**Approaches and advantages specific to the type of data to be processed and analytical needs.**

- Regression analysis forecasts a continuous output by considering one or more predictor variables [23].
- Cluster analysis methods group similar items based on shared characteristics. These algorithms help identify patterns within the data [24].
- Support vector machine (SVM) categorizes data by identifying the optimal boundary that divides distinct groups [25].
- Decision trees analyze data by using a series of questions and rules, resulting in the generation of predictions or classifications [26].
- Random forest (RF) uses a set of decision trees to enhance predictive precision and mitigate overfitting, a phenomenon in which predictions are accurate for training data but not for new data [27].
- Bayesian networks model the relationships and dependencies among variables using probability theory [28]. They are represented through a directed acyclic graph. This approach facilitates the prediction of outcomes based on established conditions.
- Markov models represent the transitions between states in a system using probabilities [29]. They are characterized by the Markov property, where the future state depends only on the current state and not on the sequence of events that preceded it.
- Fuzzy systems are based on fuzzy logic, which extends classical Boolean logic to handle the concept of partial truth, where truth values can range between completely true and completely false [30].
- Neural networks (NNs) are inspired by the human brain. They rely on interconnected nodes to process data and detect connections [31]. This model can be subdivided based on its specific use.
  - Convolutional neural networks (CNNs) process data that displays a grid-like structure, such as images [32].
  - Recurrent neural networks (RNNs) predict sequences [33]. They use their internal state (memory) to process sequences of inputs, such as language or time series data.
  - Long short-term memory (LSTM) networks are a type of RNN that can learn long-term dependencies [34]. They are ideal for activities that require comprehension of long sequences.
  - Deep neural networks (DNNs) consist of multiple interconnected layers of neurons [35]. These networks can learn from extensive amounts of data and detect complex patterns.
  - Transformers are a type of network that relies on self-attention mechanisms, allowing it to weigh the importance of different parts of the input data [36].
  - Large language models (LLMs) are advanced types of networks that have been trained on vast datasets of words and sentences [37]. They produce coherent, human-like responses to written text by selecting the most probable next words.

These AI models highlight the potential of this technology in educational contexts. The United Nations Educational, Scientific and Cultural Organization indicates that digital technologies have the potential to complement, enrich, and transform education, aligning with the United Nations’ Sustainable Development Goal 4 (SDG 4) for education and providing universal access to learning [38]. Consequently, the integration of AI in surgical training could boost independence, self-study, engagement, and motivation.

## Methods

### Overview

This review adheres to the PRISMA-ScR (Preferred Reporting Items for Systematic Reviews and Meta-Analyses extension for Scoping Reviews; see Multimedia Appendix 1) statement, designed for publications in the health and medical sciences [39]. The review process was organized following a structured protocol consisting of four stages: (1) planning, which involved establishing the criteria for the search and databases to be used; (2) conducting, which entailed performing the search and applying filters for the scoping review; (3) reporting, which included compiling the studies that met the criteria and were included in the review. During stages 1 and 2, the research papers were compiled, and the initial screening process was conducted, focusing solely on papers that fall within the scope of the review and were published in peer-reviewed scientific journals. Stage 3 consists of identifying the main characteristics that distinguish the contributions and unique features of each article that has passed the initial screening process. Subsequently, the necessary analysis was performed to present the summary of the research and compile tables and figures. The starting date for stages 1 and 2 of the scoping review was February 27, 2024, and it concluded on March 18, 2024.

### Information Sources

A total of 3 databases were selected to search for relevant studies: PubMed, Scopus, and Web of Science. The inclusion of Web of Science and Scopus databases consolidates information from other sources, such as IEEE Xplore, ScienceDirect, and SpringerLink. Therefore, they expand the scope of accessible academic literature. These platforms also provide search and analytical tools, making it easier to find pertinent studies and analyze trends. By using the 3 databases, the review considered articles with different AI models beyond the limitation of just focusing on clinical trials. By implementing this procedure, the scope of the review is expanded, enabling the identification of significant manuscripts to identify areas of opportunity in the field.

### Search Strategy

A total of 4 keywords related to AI concepts and 4 keywords related to surgical training were selected based on the research questions. The selected keywords were converted into search strings and processed to be compatible with the advanced search tool of each database. Table 1 shows the search strings used in this scoping review.

**Table 1:** Search strings used in the advanced search tools of PubMed, Web of Science, and Scopus.

Database	String of keywords
PubMed	(“Artificial Intelligence”[MeSH] OR “AI” OR “machine learning” OR “deep learning”) AND (“Surgical Training” OR “surgical education” OR “surgical assessment” OR “surgical evaluation”)
Web of Science	TS = ((“artificial intelligence” OR “AI” OR “machine learning” OR “deep learning”) AND (“surgical training” OR “surgical education” OR “surgical assessment” OR “surgical evaluation”))
Scopus	(TITLE-ABS-KEY(“artificial intelligence” OR “AI” OR “machine learning” OR “deep learning”) AND TITLE-ABS-KEY (“surgical training” OR “surgical education” OR “surgical assessment” OR “surgical evaluation”))

### Eligibility Criteria

Records retrieved from the initial search were examined to verify their compliance with the eligibility criteria and their alignment with the research questions (Textbox 2).

**Eligibility criteria.**

The inclusion criteria for this review were limited to:

- Studies published from January 2020 to March 2024 were reviewed to ensure the review covers the most recent advancements in artificial intelligence (AI) applications in surgical training.
- Full-text articles available in English to allow thorough review and analysis.
- Studies that focus on the application of AI in surgical training and evaluation, aligning with the research questions.

For the exclusion criteria, this review applied the following criteria:

- Studies not centered on the application of AI to assess or evaluate surgical training.
- Nonscientific journal publications, non–full-text articles available online, and preprints.

### Data Charting and Synthesis

After the inclusion and exclusion criteria had been applied during screening, data were charted for each included study covering three dimensions: (1) the surgical procedure (eg, laparoscopy, minimally invasive surgery, neurosurgery, and arthroscopy), (2) the AI model (eg, support vector machine [SVM], convolutional neural network [CNN], deep neural network [DNN], long short-term memory [LSTM], and transformers), and (3) the training setup (eg, simulation platforms, box trainers, surgical video analysis, in-vivo settings, virtual reality, and da Vinci system). These variables structured the subsequent evidence synthesis and guided the organization of results by procedure, technique, and setup. In addition, bibliographic fields, including year of publication and type of publication, were also charted to support descriptive reporting in the Results section. This structured approach enabled a descriptive and narrative synthesis aimed at elucidating how AI contributes to educational outcomes and skill acquisition in surgical training.

## Results

### Search Results and Study Selection

**Figure 1:**
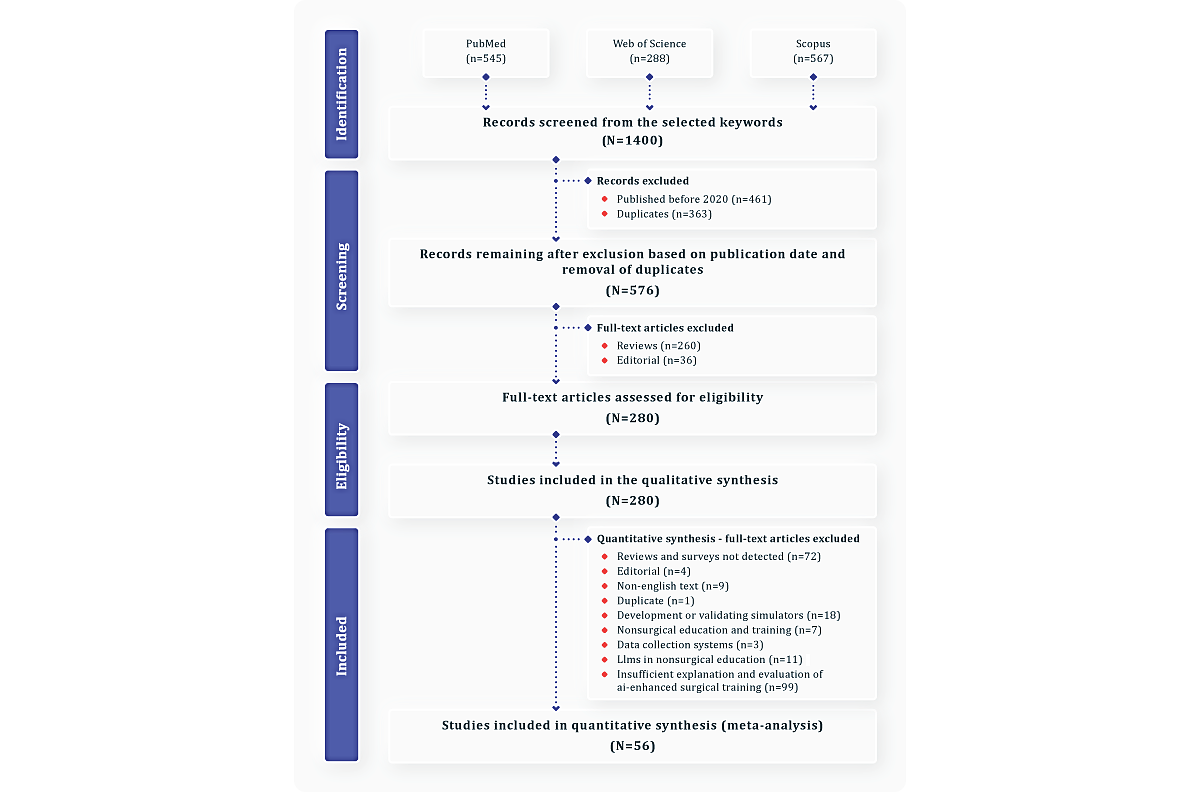
Flow diagram of the scoping review process, illustrating the inclusion and exclusion criteria. AI: artificial intelligence; LLM: large language model.

Figure 1 presents the PRISMA-ScR flow diagram illustrating the complete selection process. The initial search identified 1400 records: 545 from PubMed, 288 from Web of Science, and 567 from Scopus, obtained using the search strings described in Table 1. After applying the publication date range from January 2020 to March 2024, a total of 461 records were excluded, leaving 939 for further screening. Duplicate removal eliminated 363 records, yielding 576 unique studies.

Subsequent filtering was conducted in stages to ensure methodological rigor and relevance. Database parameters were adjusted to retain only peer-reviewed journal articles and conference proceedings, excluding 260 reviews and 36 editorials that did not meet the inclusion criteria. A total of 280 records proceeded to qualitative screening. During this stage, the relevance of each article to the review objectives was reassessed. This process excluded 76 studies that, despite meeting database filters, were secondary reviews, surveys, or editorials; 9 non-English papers, 7 papers focused on nonsurgical training, and 18 papers described simulator development or validation without AI integration. Additional exclusions comprised 1 duplicate, 3 studies addressing “Data Collection Systems,” 11 centered on “LLMs in Non-Surgical Education,” and 99 that did not provide sufficient information about AI-enhanced surgical training. This filtering process excluded 224 additional studies, leaving 56 studies for the final synthesis and analysis.

The characteristics of the 56 included studies are summarized in Table 2, organized across five domains: (1) surgical procedure (eg, laparoscopy, minimally invasive surgery [MIS], neurosurgery, and arthroscopy), (2) year of publication, (3) type of publication, (4) AI technique or model used (eg, SVM, CNN, DNN, LSTM, and transformers), and (5) training setup (eg, simulation platforms, box trainers, da Vinci system, surgical video analysis, and in vivo or virtual-reality environments). This structure enables direct comparison across specialties and methodological approaches, while supporting a descriptive and narrative synthesis of cross-cutting trends.

Across the included studies, MIS, neurosurgery, and laparoscopy represented the majority of AI applications. ML and DL techniques were the most frequently used computational approaches, while simulation environments and box trainers constituted the primary training configurations. Collectively, these trends indicate a primary emphasis on risk-managed training environments that leverage accessible kinematic and video data. However, heterogeneity in studies and limited standardization of outcome measures remain persistent challenges, underscoring the need for unified evaluation frameworks in the future.

**Table 2:** Characteristics of included studies: surgical procedures, artificial intelligence (AI) techniques, and training setups.

Classification and references			Year		Type		AI model		Setup
**MIS^a^ skills**									
	Rashidi et al [40]	2023		Journal		Fuzzy systems		Box trainer	
	Fathabadi et al [41]	2022		Conference		Fuzzy systems		Box trainer	
	Deng et al [42]	2021		Conference		CNN^b^		Box trainer	
	Kulkarni et al [43]	2023		Journal		Clustering		Box trainer	
	Wu et al [44]	2021		Journal		ML^c^ (unspecified)		da Vinci system	
	Brown and Kuchenbecker [45]	2023		Journal		Regression analysis		da Vinci system	
	Keles et al [46]	2021		Journal		ML (unspecified)		Box trainer	
	Koskinen et al [47]	2020		Journal		SVM^d^		Box trainer	
	Kasa et al [48]	2022		Journal		DL^e^ (unspecified)		Box trainer	
	Gao et al [49]	2020		Journal		Clustering		Box trainer	
	Baghdadi et al [50]	2020		Journal		Clustering		Box trainer	
	Benmansour et al [51]	2023		Journal		CNN^f^+LSTM^g^		da Vinci system	
	Yanik et al [52]	2023		Journal		CNN		Box trainer	
	Lee et al [53]	2024		Journal		Markov chains		Simulation training	
	Hung et al [54]	2023		Journal		CNN+LSTM		Simulation training	
**Neurosurgery**									
	Ledwos et al [55]	2022		Journal		Clustering		Simulation training	
	Mirchi et al [56]	2020		Journal		SVM		Simulation training	
	Yilmaz et al [57]	2024		Journal		AI (unspecified)		Simulation training	
	Siyar et al [58]	2020		Journal		SVM		Simulation training	
	Reich et al [59]	2022		Journal		NN^h^		Simulation training	
	Natheir et al [60]	2023		Journal		ML (unspecified)		Simulation training	
	Siyar et al [61]	2020		Journal		Clustering		Simulation training	
	Yilmaz et al [62]	2022		Journal		DNN^i^		Simulation training	
	Fazlollahi et al [63]	2022		Journal		Tutoring system (unspecified)		Simulation training	
	Du et al [64]	2023		Journal		SVM		Simulation training	
	Dhanakshirur et al [65]	2023		Conference		CNN		Training station	
**Laparoscopy**									
	Kuo et al [66]	2022		Journal		DL (unspecified)		Box trainer	
	Shafiei et al [67]	2023		Journal		ML (unspecified)		da Vinci system	
	Lavanchy et al [68]	2021		Journal		CNN		In-vivo setting	
	Ryder et al [69]	2024		Journal		ML (unspecified)		In-vivo setting	
	Halperin et al [70]	2024		Journal		DL (unspecified)		Box trainer	
	Ebina et al [71]	2022		Journal		SVM		Box trainer	
	Hamilton et al [72]	2023		Journal		AI (unspecified)		Training station	
	Adrales et al [73]	2024		Journal		ML (unspecified)		Surgical video	
	Wang et al [74]	2023		Conference		AI (unspecified)		Surgical video	
**Arthroscopy**									
	Mirchi et al [75]	2020		Journal		NN		Simulation training	
	Alkadri et al [76]	2021		Journal		NN		Simulation training	
	Shedage et al [77]	2021		Conference		Clustering		Simulation training	
**Ophthalmology**									
	Tabuchi et al [78]	2022		Journal		AI (unspecified)		Surgical video	
	Wang et al [79]	2022		Journal		DNN		Surgical video	
	Dong et al [80]	2021		Journal		ML (unspecified)		Surgical video	
**Robotic-assisted surgery**									
	Simmonds et al [81]	2021		Journal		Clustering		Simulation training	
	Kocielnik et al [82]	2023		Conference		DL (unspecified)		da Vinci system	
	Wang et al [83]	2023		Journal		Bayesian network		da Vinci system	
**Open surgery**									
	Bkheet et al [84]	2023		Journal		DL (unspecified)		Surgical video	
	Kadkhodamohammadi et al [85]	2021		Journal		CNN		Surgical video	
**Surgery**									
	Papagiannakis et al [86]	2020		Conference		ML (unspecified)		Simulation training	
	Thanawala et al [87]	2022		Journal		ML (unspecified)		Case logs	
**Surgery skills**									
	Sung et al [88]	2020		Journal		CNN		Simulation training	
	Khan et al [89]	2021		Journal		ML (unspecified)		Motion data	
**Otolaryngology**									
	Lamtara et al [90]	2020		Conference		ML (unspecified)		Simulation training	
**Orthopedics**									
	Sun et al [91]	2021		Journal		ML (unspecified)		Surgical video	
**Plastic surgery**									
	Kim et al [92]	2020		Conference		DL (unspecified)		Medical images	
**Radiology**									
	Saricilar et al [93]	2023		Journal		NN		Simulation training	
**Urology**									
	Kiyasseh et al [94]	2023		Journal		Transformer		Surgical video	
**Vascular surgery**									
	Guo et al [95]	2020		Journal		SVM+RF^j^		Slave controller	

^a^MIS: minimally invasive surgery.

^b^CNN: convolutional neural network.

^c^ML: machine learning.

^d^SVM: support vector machine.

^e^DL: deep learning.

^f^CNN: convolutional neural network.

^g^LSTM: long short-term memory.

^h^NN: neural network.

^i^DNN: deep neural network.

^j^RF: random forest.

### Findings and Interpretation

#### Specific Surgical Procedures

The scoping review reveals the range of surgical procedures where AI algorithms are being used (see Table 3). The analysis emphasizes the integration of AI in MIS skills (27%, 15/56) [40-54], neurosurgery (20%, 11/56) [55-65], and laparoscopy (16%, 9/56) [66-74] (see Table 3). Moderate representation was observed in arthroscopy (5%, 3/56) [75-77], ophthalmology (5%, 3/56) [78-80], and robot-assisted surgery (5%,3/56) [81-83]. Several other domains appeared less frequently, including open surgery (4%, 2/56) [84,85], general surgery (4%, 2/56) [86,87], and surgery skills (4%, 2/56) [88,89]. Finally, isolated studies were identified in otolaryngology (2%, 1/56) [90], orthopedics (2%, 1/56) [91], plastic surgery (2%, 1/56) [92], radiology (2%, 1/56) [93], urology (2%, 1/56) [94], and vascular surgery (2%, 1/56) [95].

**Table 3:** Frequency of medical fields in the included articles (N=56).

Specialty	Included articles, n (%)
MIS^a^ skills	15 (27)
Neurosurgery	11 (20)
Laparoscopy	9 (16)
Arthroscopy	3 (5)
Ophthalmology	3 (5)
Robot-assisted surgery	3 (5)
Open surgery	2 (4)
Surgery	2 (4)
Surgery skills	2 (4)
Otolaryngology	1 (2)
Orthopedy	1 (2)
Plastic surgery	1 (2)
Radiology	1 (2)
Urology	1 (2)
Vascular surgery	1 (2)

^a^MIS: minimally invasive surgery.

Functionally, most studies focused on automated skill assessment and learning-curve analysis, while comparatively few examined procedure guidance, workflow recognition, or decision support. This trend was especially evident in MIS and laparoscopy, which relied heavily on video-centric datasets and computer-vision models [40-54,66-74], and in neurosurgery, where virtual reality simulators provided standardized training environments and feedback mechanisms [55-65]. The specialty distribution appears to be driven by the availability of high-quality labeled data. Overall, the distribution of specialties indicates that AI integration aligns strongly with domains that generate structured, labeled, and reproducible data, such as endoscopic or robotic procedures. By contrast, open and specialty surgeries remain underrepresented, constrained by the limited standardization of datasets and variability in operative workflows. Future progress will depend on developing shared, procedure-specific repositories, cross-institutional benchmarks, and multimodal data capture beyond video and kinematic streams to enhance generalizability and educational impact [84-95].

#### AI Techniques Used

The scoping review identified a diverse set of AI techniques in surgical training (see Table 4). The most frequent were ML (unspecified; 21%, 12/56) [44,46,60,67,69,73,80,86,87,89-91], clustering (13%, 7/56) [43,49,50,55,61,77,81], and CNNs (11%, 6/56) [42,52,65,68,85,88]. We also observed DL (unspecified; 11%, 6/56) [48,66,70,82,84,92] and SVMs (9%, 5/56) [47,56,58,64,71], followed by neural networks (NNs; 7%, 4/56) [59,75,76,93] and AI (unspecified; 7%; 4/56) [57,72,74,78]. Additional categories included CNN+LSTM (4%, 2/56) [51,54], DNNs (4%, 2/56) [62,79], and fuzzy systems (4%, 2/56) [40,41]. Single-study categories (2%, 1/56) included regression analysis [45], Markov chains [53], tutoring system (unspecified) [63], Bayesian network [83], transformer [94], and SVM+RF [95].

**Table 4:** Application of artificial intelligence (AI) techniques in the included articles (N=56).

AI technique	Included articles, n (%)
ML^a^ (unspecified)	12 (21)
Clustering	7 (13)
CNNs^b^	6 (11)
DL^c^ (unspecified)	6 (11)
SVMs^d^	5 (9)
NNs^e^	4 (7)
AI (unspecified)	4 (7)
CNN+LSTM^f^	2 (4)
DNNs^g^	2 (4)
Fuzzy systems	2 (4)
Regression analysis	1 (2)
Markov chains	1 (2)
Tutoring system (unspecified)	1 (2)
Bayesian network	1 (2)
SVM+RF^h^	1 (2)
Transformer	1 (2)

^a^ML: machine learning.

^b^CNN: convolutional neural network.

^c^DL: deep learning.

^d^SVM: support vector machine.

^e^NN: neural network.

^f^LSTM: long short-term memory.

^g^DNN: deep neural network.

^h^RF: random forest.

From 2020 to 2024 (see Table 5), ML (unspecified) appears every year, CNNs strengthen in 2021 and 2023, and DL (unspecified) is present in 2020 and 2022-2024. Sequential and hybrid models (CNN+LSTM and DNNs) clusters in 2022-2023. AI (unspecified) emerges from 2022 onward. Probabilistic and rule-based approaches (Bayesian networks, fuzzy systems, and Markov chains) and transformer/SVM+RF appear as single-study categories. Overall, the technique mix tracks data modality and availability (video and kinematics), reinforcing the need for shared multimodal repositories and standardized evaluation metrics to compare methods fairly and improve external validity.

**Table 5:** Temporal distribution of artificial intelligence (AI) models in the included articles (2020-2024).

AI model	2020, n (%)	2021, n (%)	2022, n (%)	2023, n (%)	2024, n (%)	Total, n (%)
ML^a^ (unspecified)	2 (17)	5 (42)	1 (8)	2 (17)	2 (17)	12 (100)
CNN^b^	1 (17)	3 (50)	0 (0)	2 (33)	0 (0)	6 (100)
Clustering	3 (43)	2 (28)	1 (14)	1 (14)	0 (0)	7 (100)
SVM^c^	3 (60)	0 (0)	1 (20)	1 (20)	0 (0)	5 (100)
DL^d^ (unspecified)	1 (17)	0 (0)	2 (33)	2 (33)	1 (17)	6 (100)
NN^e^	1 (25)	1 (25)	1 (25)	1 (25)	0 (0)	4 (100)
AI (unspecified)	0 (0)	0 (0)	1 (25)	2 (50)	1 (25)	4 (100)
DNN^f^	0 (0)	0 (0)	2 (100)	0 (0)	0 (0)	2 (100)
CNN+LSTM^g^	0 (0)	0 (0)	0 (0)	2 (100)	0 (0)	2 (100)
Fuzzy systems	0 (0)	0 (0)	1 (50)	1 (50)	0 (0)	2 (100)
Bayesian network	0 (0)	0 (0)	0 (0)	1 (100)	0 (0)	1 (100)
Markov chains	0 (0)	0 (0)	0 (0)	0 (0)	1 (100)	1 (100)
Regression analysis	0 (0)	0 (0)	0 (0)	1 (100)	0 (0)	1 (100)
SVM+RF^h^	1 (100)	0 (0)	0 (0)	0 (0)	0 (0)	1 (100)
Transformer	0 (0)	0 (0)	0 (0)	1 (100)	0 (0)	1 (100)
Tutoring system (unspecified)	0 (0)	0 (0)	1 (100)	0 (0)	0 (0)	1 (100)
Total per year	12 (21)	11 (20)	11(20)	17 (30)	5 (9)	56 (100)

^a^ML: machine learning.

^b^CNN: convolutional neural network.

^c^SVM: support vector machine.

^d^DL: deep learning.

^e^NN: neural network.

^f^DNN: deep neural network.

^g^LSTM: long short-term memory.

^h^RF: random forest.

In the analyzed studies, the number of publications increased from 12 in 2020 to 17 in 2023, with 11 in both 2021 and 2022, and 5 in 2024. The literature search concluded on March 18, 2024, which likely accounts for the lower count in 2024. These totals are summarized in the “Total per year” row of Table 5.

#### Application of AI Techniques

AI techniques have been applied across diverse training setups, enhancing both learning experiences and performance assessment in surgical procedures (see Table 6). The most frequent environments were simulation training (36%, 20/56) [53-64,75-77,81,86,88,90,93] and box trainers (23%, 13/56) [40–43,46-50,52,66,70-71], followed by surgical video analysis (16%, 9/56) [73,74,78-80,84,85,91,94] and robotic systems using the da Vinci platform (11%, 6/56) [44,45,51,67,82,83]. Less frequent configurations included training stations (4%, 2/56) [65,72] and in-vivo settings (4%, 2/56) [68,69], with single-study setups for case logs [87], motion data [89], medical images [92], and a slave controller [95] (each 2%, 1/56). Across these settings, studies reported the use of automated skill assessment, formative feedback, and adaptive progression, supported by video, kinematic, and performance-metric streams.

**Figure 2:**
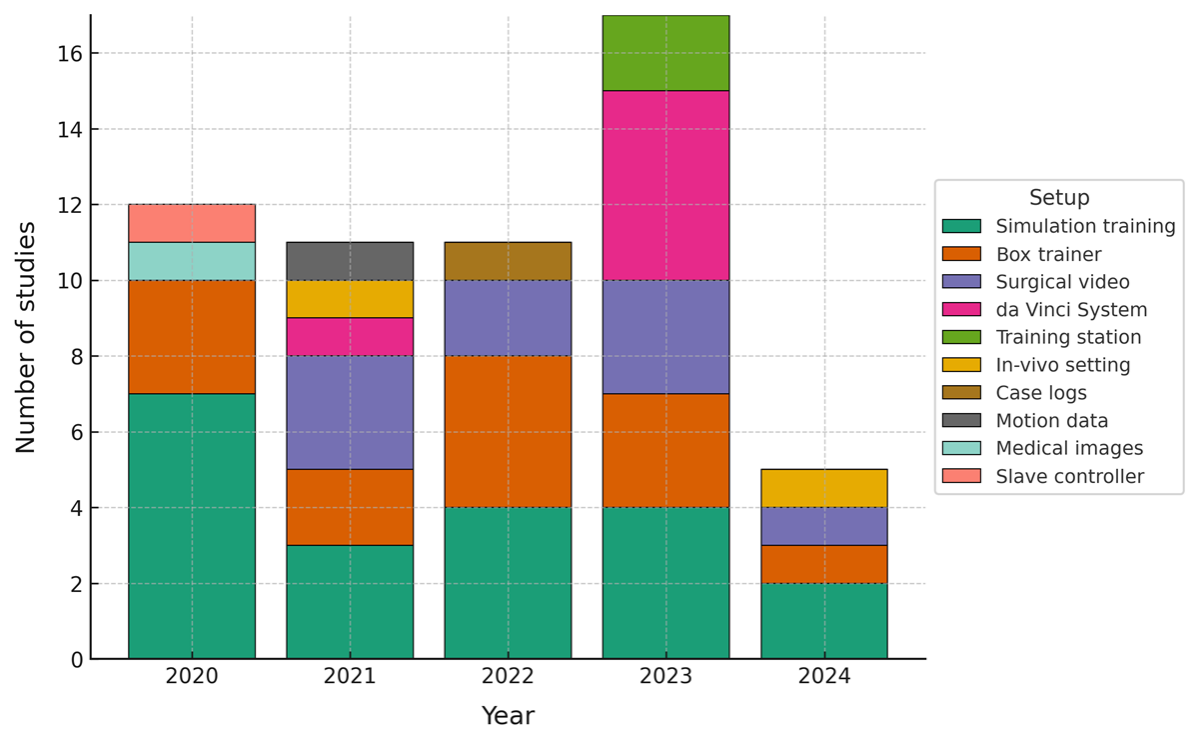
Appearance of setups over the years in the included articles.

Over time, setup diversity increased, peaking in 2023 (see Figure 2). Simulation training and box trainers were consistently present, while surgical video and da Vinci deployments clustered in 2021-2023. These patterns mirror data availability and standardization in risk-managed environments, where AI can be trained and evaluated reliably.

**Table 6:** Distribution of training setups in the included articles (N=56).

Training setup	Included articles, n (%)
Simulation training	20 (36)
Box trainer	13 (23)
Surgical video	9 (16)
da Vinci System	6 (11)
Training station	2 (4)
In-vivo setting	2 (4)
Case logs	1 (2)
Motion data	1 (2)
Medical images	1 (2)
Slave controller	1 (2)

## Discussion

### Principal Findings

This section discusses the study’s implications and contributions to the field. The review maps and analyzes current applications of AI in surgical training, assessment, and evaluation, identifying the most common surgical procedures, AI techniques, training setups, and highlighting gaps and opportunities for future research. The results show that AI is most frequently reported in data-rich, risk-mitigated environments, notably simulation training and box-trainer setups, and that ML (unspecified) and DL (unspecified) approaches dominate model choices.

Within these settings, many studies report models that leverage synchronized inputs, for example, kinematics, video, and other performance metrics, to classify technical skill using consistent criteria, to characterize learning trajectories across repeated attempts, and to localize performance-limiting behaviors at the level of gestures, steps, or procedural phases. When embedded in iterative practice, these capabilities may enable individualized training pathways that adjust task parameters and feedback density to a trainee’s evolving competence, with the potential to shorten time to proficiency and to reduce instructor workload. These implications are consistent with the results, in which simulation training accounted for 36% (20/56) and box trainer setups for 23% (13/56) of the included studies.

### Findings in Relation to the Research Questions

Regarding the first research question aimed at identifying the specific surgical procedures where AI algorithms are most frequently applied in surgical training, AI use concentrates on MIS skills [40-54], neurosurgery [55-65], and laparoscopy [66-74]. Rather than simple frequency, the common thread across these areas is structured, high-signal data capture and well-specified tasks. Endoscopic and robotic workflows generate synchronized video, robotic kinematics, and simulator logs, which enable reproducible labels such as phase boundaries, gesture events, and Objective Structured Assessment of Technical Skills–aligned rubrics. This ecosystem lowers barriers to annotation and validation, thereby accelerating method development. Beyond these clusters, activity in ophthalmology [78-80], open surgery [84,85], robot-assisted surgery [81-83], and single-study specialties including radiology [93], urology [94], and vascular surgery [95] signals a widening scope. However, these domains often face less standardized capture or a more variable field-of-view, which complicates model training and external validation. The overall distribution, therefore, appears to reflect data tractability and curricular formalization more than inherent differences in educational need.

The second research question investigated which AI techniques have been used in surgical training and evaluation. Studies use ML (unspecified) [44,46,60,67,69,73,80,86,87,89-91] and DL (unspecified) [48,66,70,82,84,92] as broad families, with task-appropriate specializations such as CNNs for video [42,52,65,68,85,88] and SVMs for lower-dimensional kinematics or hand-crafted features [47,56,58,64,71]. NNs [59,75,76,93] support competency modeling when feature engineering is feasible, and CNN+LSTM hybrids [51,54] target temporal dynamics for suturing and task segmentation. DNNs are explicitly mentioned in [62,79]. Single-study categories (fuzzy systems [40,41], regression analysis [45], Markov chains [53], tutoring system (unspecified) [63], Bayesian network [83], transformers [94], and SVM+RF [95]) illustrate exploratory breadth rather than established consensus. Consistent with coding, CNN+LSTM is treated as a distinct class and not double-counted under CNNs. No single approach emerges as universally optimal; instead, methods align with task structure (classification vs sequence prediction), signal characteristics (video and kinematics), and assessment granularity (summative scores versus frame- or gesture-level feedback).

The third research question investigated how AI techniques are being used to assess and improve surgical training. Across setups, a common pattern is the move from retrospective, manual scoring to prospective, automated analytics that are both standardized and timely. In simulation training, synchronized streams enable immediate feedback and progression gating, which supports deliberate practice cycles grounded in objective metrics. This aligns with the preponderance of simulation studies in the dataset and the consistent application of ML and DL to transform kinematics and video into competency-linked outputs. In box trainers, models quantify motion economy, tool path quality, and task efficiency, enabling skill stratification and targeted coaching [40-43,46-50,52,66,70,71]. In robotic systems on the da Vinci platform, studies demonstrate automated assessment, uncertainty-aware feedback, and domain adaptation for cross-site or cross-task transfer [44,45,51,67,82,83]. In surgical video pipelines, investigators focus on procedural understanding, ergonomics, and fine-grained performance analytics [73,74,78-80,84,85,91,94]. The unifying mechanism across these contexts is measurement at scale that reduces feedback latency, increases consistency, and enables adaptive progression rules without displacing instructor oversight.

Finally, the last research question investigated the way in which AI applications in surgical training affect the learning curve of surgical residents and fellows. Multiple studies report outcomes consistent with accelerated learning and improved technical performance under AI-enabled training. This includes predictive modeling of progression [49], metric selection and learning-curve characterization in simulation [55], a randomized comparison of feedback modalities [57], competency-based training backed by neural models [59], continuous monitoring of bimanual expertise with deep models [62], and competency estimation in laparoscopic training [69]. Evidence from robotic contexts shows that automated assessment can structure practice with short feedback loops [45]. That said, effect sizes remain difficult to aggregate due to heterogeneous study designs, small sample sizes, nonstandard outcome measures, and limited external validation. The most defensible interpretation is that personalized, data-driven feedback and objective, repeated measurement are plausible mechanisms for the observed gains, with further multicenter validation needed to establish generalizability and durability.

The findings suggest that current AI deployment in surgical training follows data availability and standardization, that ML/DL with video and kinematics are dominant because they best match that data, and that automated, timely feedback is the primary lever through which AI influences performance and learning. Where capture is less standardized or external validation is sparse, adoption tends to lag. This synthesis directly motivates the recommendations presented later in the Discussion section on common benchmarks, transparent reporting, and SDG 4–aligned scalability.

### Comparison With Previous Work

Systematic literature reviews in surgical training found in the literature have focused on specific training methods (eg, simulation-based training) or on specific types of surgery (eg, plastic surgery and orthopedic surgery) rather than providing a cross-specialty map of AI methods for training, assessment, and evaluation. Reviews focused on simulation-based training within specific domains underscore this pattern. Lawaetz et al [96] examined simulation-based training and assessment in open vascular surgery, cataloguing common methods and commenting on effectiveness within that context. Abelleyra Lastoria et al [97] surveyed simulation-based tools in plastic surgery and concluded that the validity of many approaches requires further investigation. Woodward et al [98] reached a similar conclusion in orthopedic surgery, noting concerns about the construct validity and methodological rigor of simulation studies. Reviews centered on robotic-assisted surgery also reflect divergent emphases: Rahimi et al [99] provided a descriptive overview of training modalities and assessment practices, whereas Boal et al [100] explicitly scrutinized AI methods for technical skills in robotic surgery and highlighted that both manual and automated assessment tools are often insufficiently validated.

Closer to the scope of the present scoping review, several analyses have examined automation and AI across surgical training tasks. Levin et al [101] identified families of automated technical skill assessment methods, including computer vision, motion tracking, ML and DL, and performance classification, but did not synthesize evidence on educational effectiveness. Lam et al [102] focused specifically on ML methods and reported accuracy rates that generally exceeded 80 percent across included studies, offering a performance-oriented view rather than a training-context analysis. Pedrett et al [103] emphasized the central role of video-derived motion and robotic kinematic data as inputs to AI models for technical skill assessment in minimally invasive surgery, reinforcing the importance of structured, high-signal data streams.

Findings from the present review are consistent with these previous observations in several respects. First, the centrality of simulation and other risk-managed environments recurs across literature, reflecting where ground truth is tractable and measurement can be standardized. Second, many reviews identify validation gaps, noting that reported metrics, dataset partitions, and labeling practices vary widely, which complicates comparison across sites and inhibits external generalizability [96-100]. Third, there is broad agreement that AI-assisted assessment is advancing rapidly in robotic and minimally invasive settings; yet, many frameworks remain descriptive or single-center, and their educational impact is not consistently established with robust designs [99-103].

At the same time, this review differs from earlier work in several ways. The scope extends across specialties and across training setups, linking procedures, techniques, and use cases in a single comparative framework. Rather than isolating a single algorithm family or specialty, the analysis connects the dominant AI techniques to the data modalities they exploit and to the assessment functions they serve. This mapping clarifies why ML and DL approaches, particularly CNN-based and hybrid temporal models, are prevalent where high-quality video and kinematics are available, and why adoption is slower where capture is less standardized. In addition, the review integrates signals relevant to learning curves, highlighting studies that associate AI-enabled feedback with improvements in proficiency trajectories, while also acknowledging heterogeneity and the need for external validation. By taking this comparative perspective, the review identifies shared deficiencies that cut across specialties, including nonstandard outcome measures, limited transparency in algorithmic reporting, and sparse multicenter testing, and points toward future work on benchmarks, interoperable data schemas, and scalable deployment aligned with SDG 4.

Whereas previous reviews have been primarily domain-specific or method-specific, this scoping review offers a cross-specialty synthesis that links where AI is used, which techniques are used, and how they are used to support training and assessment. This perspective complements existing literature by emphasizing comparability across contexts, illuminating mechanisms by which AI influences learning, and articulating the methodological steps needed to translate promising prototypes into reproducible, generalizable, and educationally meaningful tools.

### Strengths and Limitations

This scoping review offers a broad, cross-specialty perspective on the application of AI in surgical training, assessment, and evaluation. It maps procedures, techniques, and training setups within a single comparative framework, which supports interpretation across contexts rather than within a single specialty. The review adheres to PRISMA-ScR guidance, applies explicit inclusion and exclusion criteria, and uses transparent counting rules that assign each study a primary AI technique and a primary setup to avoid double-counting. Results are presented as both narrative synthesis and structured summaries. The Discussion integrates an SDG 4 perspective, offering concrete implementation considerations related to access, scalability, and equity. Together, these elements provide a panoramic view of where AI is currently deployed, why certain methods dominate in specific data environments, and how these choices influence assessment and feedback in practice.

Several constraints should be considered. First, the search was limited to English-language publications and to the period ending March 18, 2024, which may omit relevant work outside this window. Second, many articles describe methods only at a general label level (AI, ML, and DL) without specifying architectures or training details, which limits interpretability and reproducibility. Third, the evidence base is concentrated in simulation, box-trainer, and video-centric settings, which may not fully capture transfer to live clinical performance, patient outcomes, or longer-term retention. Fourth, external validation is limited, as relatively few studies report multicenter testing, performance under domain shift, subgroup analyses, or calibration, which constrains confidence in portability.

To address these limitations, educational outcomes should also be mapped to recognized competency frameworks and reported with standardized metrics that enable replication and meta-synthesis. When multisetup or multi-technique pipelines are used, authors should specify proportional attribution. Reporting on access, resource requirements, and cost per trainee hour will support the deployment and equity assessment of SDG 4. Multicenter collaborations that release shared benchmarks and interoperable datasets will be necessary to improve reproducibility and to allow fair comparisons across techniques and settings.

### Future Work Recommendations

This scoping review identified current applications of AI in surgical education and highlighted priority areas for further work. As summarized in Table 6 and visualized in Figure 2, a large proportion of studies focus on simulation training [53-64,75-77,81,86,88,90,93], representing 36% (20/56) of the included articles. This concentration reflects the suitability of simulation for controlled data capture and iterative practice. Building on this foundation, AI can enhance simulation-based training with realistic, adaptive, and personalized learning experiences [104,105], while also enabling standardized and rapid feedback that supports deliberate practice.

Advances in computer vision are particularly significant where high-quality video and kinematic data are accessible, which aligns with the prevalence of simulation and box-trainer studies in the included literature. In these regulated, risk-mitigated environments, AI systems can produce timely and structured feedback linked to defined competency frameworks, including economy of motion, bimanual coordination, camera control, tissue handling, and ergonomics, thereby facilitating deliberate practice. Although natural language processing technologies are less represented in the current review, their growing maturity suggests near-term opportunities to integrate narrative guidance, rubric-based feedback, and reflective prompts alongside quantitative metrics, provided such outputs are aligned with curricular objectives and are appropriately validated.

Future efforts should pursue 5 complementary directions.

First, strengthen external validity. Studies should include multi-institution cohorts, predefined external test sets, and reporting of performance under domain shift, including different camera views, instruments, and case difficulty. Where feasible, researchers should evaluate the transfer from simulation or bench-top tasks to higher-fidelity or clinical settings with clearly specified outcome measures and follow-up intervals.

Second, standardize educational outcomes. Investigators should map AI outputs to recognized competency frameworks and report validity, reliability, learning curve parameters, and time to competency with consistent definitions. Agreement on core outcome sets will enable comparison across techniques and facilitate meta-synthesis.

Third, expand the breadth and transparency of data. New work should prioritize multimodal capture that combines video, kinematics, tool telemetry, where appropriate, eye tracking or physiological signals. Public or data-sharing consortia should release interoperable schemas, labeling protocols, and benchmark tasks that are specific to procedures and skill elements. Clear descriptions of models and training and validation splits will improve reproducibility.

Fourth, improve usability, equity, and scalability in alignment with SDG 4. Models should operate on standard hardware, interoperate with existing simulators and video platforms, and function reliably in low-bandwidth or offline environments. Reporting of access, installation steps, resource needs, and cost per trainee hour will support adoption in diverse settings. Interfaces should disclose uncertainty, make feedback interpretable, and integrate into educator workflows without adding undue burden.

Fifth, broaden methodological scope responsibly. There is an opportunity to study natural language technologies for rubric-based guidance, structured debriefs, and reflective prompts, provided outputs are aligned with curricular objectives and validated for educational use. Prospective trials that compare feedback modalities and density, and that measure downstream retention and transfer, will clarify how AI should be integrated pedagogically.

Together, these directions could move the field from promising prototypes toward reproducible, generalizable, and educationally meaningful tools that improve surgeon training while supporting equitable access to high-quality education.

### Conclusions

This scoping review maps current applications of AI in surgical training, assessment, and evaluation across procedures, techniques, and training setups. From 1400 records, 56 studies met the inclusion criteria, with activity concentrated in minimally invasive surgery, neurosurgery, and laparoscopy. AI is most frequently deployed in data-rich, risk-mitigated environments, particularly simulation training and box trainers, where synchronized video and kinematic streams support objective measurement and timely feedback. Technique choices reflect these data conditions, with ML (unspecified) and DL (unspecified) methods predominating and task-specific variants, such as CNNs and hybrid temporal models, applied to video-centric problems.

Across settings, studies describe automated skill assessment, structured formative feedback, and adaptive progression, with several reporting improvements consistent with accelerated learning curves. At the same time, heterogeneity in study design, small samples, nonstandard outcome measures, and limited external validation constrain strong inferences about effect sizes and generalizability. The evidence, therefore, supports cautious optimism that AI-enabled feedback can enhance skill acquisition, while underscoring the need for more rigorous evaluation.

Future work should prioritize precise reporting of models and datasets, multicenter validation, and standardized educational outcomes linked to recognized competency frameworks. Interoperable data schemes, shared benchmarks, and transparent methods will be essential to enable comparison across sites and techniques. Attention to scalability, access, and usability will support alignment with SDG 4, ensuring that benefits extend beyond well-resourced centers. With these elements in place, AI has the potential to deliver reproducible, equitable, and educationally meaningful gains in surgical training.

## Appendix

Multimedia Appendix 1 PRISMA-ScR checklist.

## Abbreviations

AI: artificial intelligence
CNN: convolutional neural network
DL: deep learning
DNN: deep neural network
LSTM: long short-term memory
MIS: minimally invasive surgery
ML: machine learning
NN: neural networks
PRISMA-ScR: Preferred Reporting Items for Systematic Reviews and Meta-Analyses extension for Scoping Reviews
SDG: Sustainable Development Goal
SVM: support vector machine

## Data Availability

The datasets generated or analyzed during this study are available in the AI Review – Selected Zotero group library [106].
